# Actor and Partner Effects of Touch: Touch-Induced Stress Alleviation Is Influenced by Perceived Relationship Quality of the Couple

**DOI:** 10.3389/fpsyg.2021.661438

**Published:** 2021-04-13

**Authors:** Difei Liu, Yi Piao, Ru Ma, Yongjun Zhang, Wen Guo, Lin Zuo, Weili Liu, Hongwen Song, Xiaochu Zhang

**Affiliations:** ^1^School of Education, Hefei University, Hefei, China; ^2^School of Humanities and Social Science, University of Science and Technology of China, Hefei, China; ^3^Institute of Advanced Technology, University of Science and Technology of China, Hefei, China; ^4^School of Life Sciences, University of Science and Technology of China, Hefei, China; ^5^School of Foreign Languages, Anhui Jianzhu University, Hefei, China; ^6^Mental Health Education Center, Southwest University of Science and Technology, Mianyang, China; ^7^Department of Radiology, the First Affiliated Hospital of USTC, Hefei National Laboratory for Physical Sciences at the Microscale and School of Life Science, Division of Life Science and Medicine, University of Science and Technology of China, Hefei, China; ^8^Academy of Psychology and Behavior, Tianjin Normal University, Tianjin, China; ^9^Hefei Medical Research Center on Alcohol Addiction, Affiliated Psychological Hospital of Anhui Medical University, Hefei Fourth People's Hospital, Anhui Mental Health Center, Hefei, China

**Keywords:** stress, relationship quality, touch, actor and partner effects, well-being

## Abstract

Because of the impact of close partner's touch on psychological and physical well-being by alleviating stress, it is important to explore the influence factors that underlie the stress-alleviating effect of close partner's touch. Previous studies suggested that the stress-alleviating effect was different when individuals were touched by different persons. Specifically, the stress was reduced significantly when the individual was touched by the close partner compared with the acquaintance and the stranger. However, whether the stress-alleviating effect of touch was modulated by the close relationship quality is unknown. To examine this question, the participants (*n* = 61) performed a 3 (i.e., alone, partner no-touch, and partner touch) × 2 (i.e., safety and threat) within-subjects experiment. The results revealed that the stress of the participants alleviated significantly while close partners present with touch compared with without touch during facing a threat. We also found that the relationship quality of couple-members (i.e., participants perceiving the quality of alternatives and the partners' commitment level) modulated touch-induced stress alleviation. Participants perceiving the low quality of alternatives and the high partners' commitment level showed stronger touch-induced stress-alleviating effect than participants perceiving the high quality of alternatives and the low partners' commitment level. The explained variance was around 16.8% jointly for actor and partner effects. These findings provide evidence for explaining the reasons for touch-induced alleviating stress and have important implications for predicting the future effect of interactive behaviors.

## Introduction

It is important to alleviate the negative impact of stress on people's health because a higher risk of diseases is linked to stress (Cohen et al., 2012). A previous research indicates that affectionate touch may buffer the potential negative impact of stressful events (Jakubiak and Feeney, 2019). Touch can convey different social intentions and messages, such as support, affiliation, dependence, and hostility (Hertenstein et al., 2006a). Touch also has important social and affective values (Löken and Olausson, 2010), especially in close relationships (Jakubiak and Feeney, 2017). It has been shown that receiving positive affectionate touch promotes the development of secure attachment, interpersonal relationships, and psychological and physical well-being (Gallace and Spence, 2010; Devine et al., 2020). Given the powerful consequence of touch for psychological and physical health, researchers have long been interested in exploring how touch alleviates stress. However, relatively few studies have focused on the predictors of the touch-induced stress-alleviating effect.

Relationship quality is a complex and multifaceted concept with far-reaching social implications (Finkel et al., 2017). In the most general sense, relationship quality refers to the subjective perception about the negative or positive relationship statue (Beckmeyer et al., 2018). A growing number of research studies attempt to explain and predict relationship quality, and there have been many self-report predictors of relationship quality (e.g., attachment style, commitment, support, alternative, satisfaction) (Joel et al., 2020). Compared with other predictors, commitment and perceived quality of available alternatives are the powerful predictors of the stability of a romantic relationship (Kelley and Thibaut, 1978; Rusbult et al., 2001), and partner's commitment may be the important factor influencing individual perceiving of relationship stability. Hence, the commitment level of the partner and perceived quality of available alternatives were used to assess romantic relationship quality in the current study. The low quality of people's close relationships was associated with bad emotion (Cano et al., 2004), poor physical (Bookwala, 2005) and psychological health (Robles et al., 2014), and meant a decrease in individual resources. According to the Conservation of Resources (COR) theory, a decrease of resources related to the decline of close relationship quality triggers stress that can cause many negative outcomes (Hobfoll, 2001). Previous studies suggested that the stress-alleviating effect was different when individuals were touched by different persons who provided them with different resources. Compared with the acquaintance and the stranger, the stress was reduced significantly when the individual was touched by close partners (Goldstein et al., 2018; Morriss et al., 2019). It seems reasonable to assume that the higher the relationship quality of the couple, the stronger touch-induced the stress-alleviating effect will be, in both the individual and the partner. However, whether the touch-induced stress-alleviating effect is connected with the relationship quality of the couple remains unknown. Specifically, it is not clear whether individuals who perceive high relationship quality have stronger stress alleviation effect and, importantly, whether the partners perceive high relationship quality at the same time.

Here, we proposed two hypotheses, and we first hypothesized that the participants would report greater declines in stress when they received an affectionate touch from their close partners than when they received no touch (Hypothesis 1). Moreover, we hypothesized that touch-induced stress alleviation would be related to the relationship quality of couple-members and the relationship quality of both sides predicted the effect of touch-induced stress alleviation (Hypothesis 2). To test these hypotheses, 61 participants performed a 3 (i.e., alone, partner no-touch, and partner touch) × 2 (i.e., safety and threat) within-subjects experiment with their partners. By comparing the stress alleviation under partner touch condition while facing a threat with the stress alleviation under partner no-touch condition while facing a threat, the current study examined the role of close partners' touch in alleviating stress. Through multiple linear regression, we further examined whether the touch-induced stress-alleviating effect was modulated by the relationship quality of couple-members.

## Materials and Methods

### Participants

The participants were recruited from the universities through advertisements posted on the campus Bulletin Board System (BBS). The participants were told that the study was about pain, and that they would go to the laboratory with their close partners for about twice. They were given no information about the effect of affectionate touch.

In previous research studies, compared with the acquaintance and the stranger, close partner's affectionate touch had a significant effect in relieving stress or pain while facing a threat (Coan et al., 2006, 2017). According to the average effect size of previous studies (effect size Cohen's *f* = 0.5), G-power was used to determine the appropriate sample size (effect size Cohen's *f* estimated at 0.50 to achieve 80% power, the required sample size was 18). To improve the reliability of the results, 64 participants (32 males and 32 females) took part in the experiment, aged from 18 to 25 years (*M*_age_ = 21.6 years, *SD*_age_ = 1.4 years). Three participants (two females and one male) were dropped from the analysis because the recordings of their self-report were always the same in the experiments.

The participants were screened by the following criteria: (i) right-handedness, (ii) no history of neurological disorders and mental illnesses, (iii) no medication used, (iv) no chronic or acute pain, (v) not pregnant, and (vi) in a heterosexual romantic relationship lasting more than 6 months. The partners in the experiment were their close partners. The study protocol was approved by the Ethics Committee of the University of Science and Technology of China. The written consents were signed by the participants, and every participant was paid 100 yuan for participation.

### Procedure

During the first visit, the eligible participants came to the laboratory with their close partners. The experimenter introduced the tasks to them. Then, couple-members were assigned to different rooms to complete the questionnaire survey [demographic questionnaire and the Investment Model Scale (IMS)]. After finishing the questionnaire survey, the participants and their partners underwent pain familiarization and pain calibration, respectively. The participants received electric shocks to the left dorsal lower arm, each administered for 20 ms in ascending or descending order with 10 s interval. The participants were asked to report the pain intensity with the numerical pain scale (NPS), ranging from 0 to 100, denoting “no pain” to “the worst pain imaginable.” At last, the stimulus intensity, which evokes a pain magnitude of 70/100 (pain-70) on the NPS for three times, was chosen for every stimuli-target. Pain-70 intensities ranged from 3.0 to 3.8 mA, with an average of 3.41 mA and *SD* of 0.23.

Then, the participants and their partners came to the laboratory again to do the formal experiment after 2 or 3 days. They were brought to the experimental room and were asked to express their feelings and emotions naturally and not to talk with each other during the experiment. The participants who were subjected to the threat of electric shock (stimuli-targets) performed a 3 (i.e., alone, partner no-touch, and partner touch) × 2 (i.e., safety and threat) within-subjects experiment. The participants were instructed to rate their agitation and unpleasantness of the anticipation stage with an 11-point scale at every trial. Concurrently, their partners were asked to focus on the participants and rate the participants' level of unpleasantness and agitation. Both the participants and their partners rated the feelings in their non-dominant hand (left hands), and the right hands were used for affectionate touch (Goldstein et al., 2018). The difference with previous studies that highlighted touch-induced analgesia effects is the ratings of unpleasantness (agitation) about the anticipation stage, instead of the perception stage. Stress arises when one appraises a situation as a threat or demand on him and does not have adequate coping resource (Cohen and Wills, 1985). Because of the uncertainty of the threat cue (indicating a 25% likelihood of receiving an electric simulation), the electric shocks in the study were presented as the forthcoming threat. The study focuses on the stress, not the pain, so these ratings were all about the perceptions in the anticipation stage.

### Experimental Conditions

The experiment included three conditions (alone, partner no-touch, and partner touch), each lasting about 12 min. A 10 min break separated every condition. During alone condition, the participants who were the stimuli-targets did the experiment in the room alone, holding the handles of the armchair by their dominant hands; meanwhile, their partners sat in another room. In partner touch condition, the participants and the partners sat face-to-face with hands-holding. While during partner no-touch condition, the participants and the partners sat face-to-face without hands-holding.

**Figure 1:**
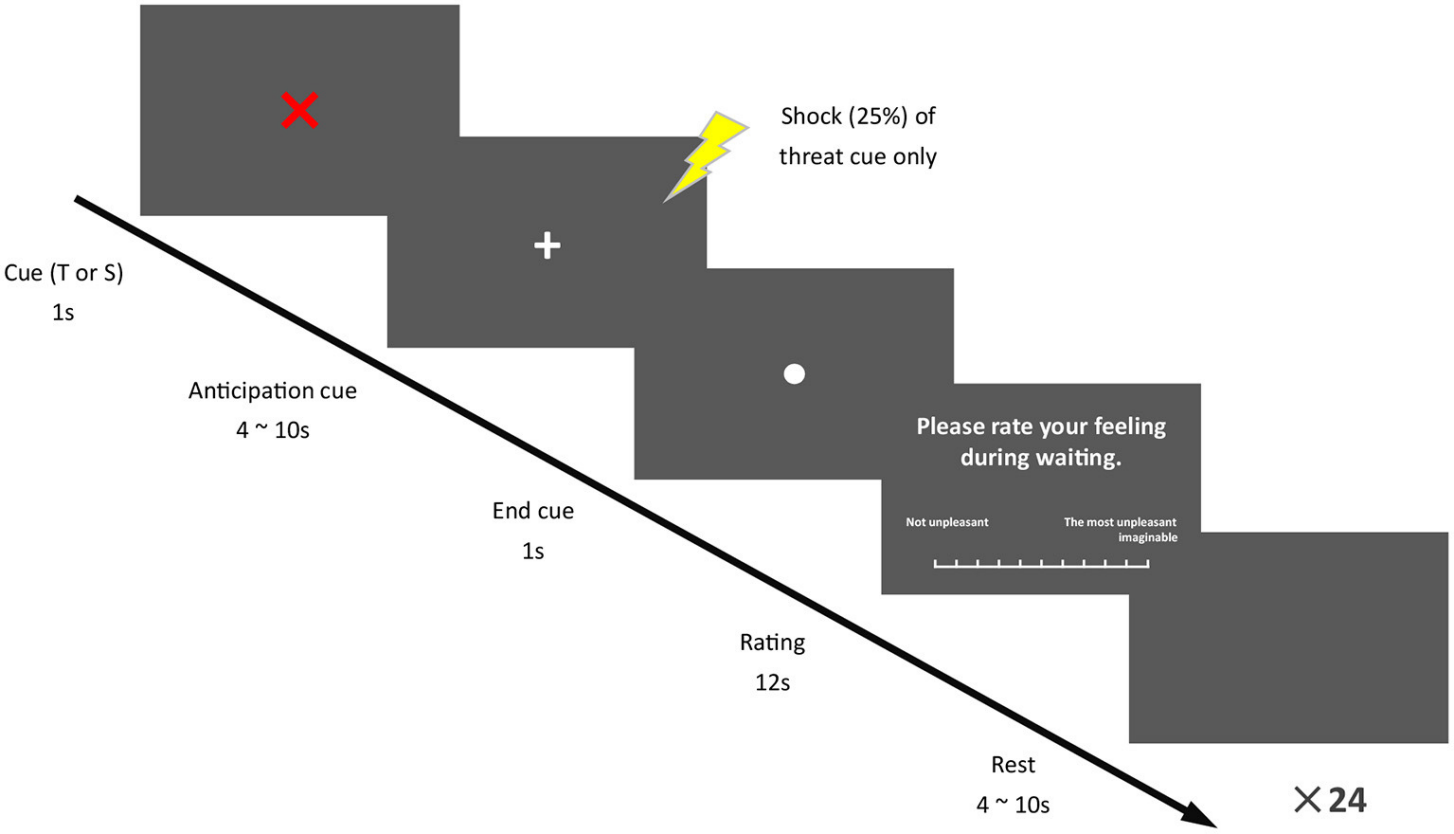
Experimental procedure. Trials were composed of five phases, 1 s T or S cue, 4–10 s anticipation phase, 1 s end cue, 12 s rating phase, and 4–10 s resting phase.

Each condition consisted of 24 trials. The trails were randomized within participants, and the condition order was counterbalanced between participants. The trial began with a threat cue or a safety cue (12 threat cues and 12 safety cues were presented in random order). Threat cue was a red “X” on a black background, indicating a 25% likelihood of receiving an electric stimulation to the left arm. Safety cue, which indicated no shock, was a blue “O” against a black background. The cue lasted for 1 s and was followed by a fixation cross, indicating a 4–10 s anticipation phase. At the end of the anticipation phase, electric stimulation might be delivered. Electric stimulation was produced by a separate physiological stimulator (Custom design, the maximum output voltage was +/−18 V, the peak-to-peak current range was adjustable continuously from 0 to 4 mA). Electric stimulation lasted for 20 ms at the current of pain-70 intensity. The ending cue of the anticipation phase was a dot. After that, the participants who were the stimuli-targets rated subjective perception of agitation (arousal) and unpleasantness (valence) during the anticipation phase by an 11-point scale. Meanwhile, the partners rated the level of agitation and unpleasantness of the stimuli-targets. The rating phase lasted for 12 s. At the end of each trial, the resting phase was presented with a black screen, varying from 4 to 10 s (Figure 1). All the participants received three or four shocks every condition (Coan et al., 2006).

### Measures

#### Arousal and Valence

The couples were instructed to choose the rating numbers on the 11-point scale to rate the level of agitation and unpleasantness during the anticipation phase. The participants who were stimuli-targets rated their agitation (i.e., arousal) and unpleasantness (i.e., valence). In the meantime, the partners rated the level of stimuli-targets' agitation and unpleasantness according to their perception. The 11-point scale for agitation (unpleasantness) was rated with “calm” (“not unpleasant”) at the left and “agitation” (“the most unpleasant imaginable”) at the right (from 0 to 10). The purpose of asking the partners to focus on the stimuli-targets and rate the stimuli-targets' perception was to ensure that they were attentive. Only the agitation (unpleasantness) ratings of the stimuli-targets were analyzed in the current study.

#### Self-Report Psychometric Measure

In addition to the demographic information, all participants and their partners completed the IMS (Rusbult et al., 1998). The IMS includes four subscales (i.e., Satisfaction, Quality of Alternatives, Investment Size, and Commitment), with seven items for Commitment and five items for three other subscales. Satisfaction measures the degree to which a person is pleased with the close relationship (“My relationship is much better than other's relationships,” “I feel satisfied with our relationship.”); Quality of Alternatives measures the degree to which the partner can be replaced by someone attractive (“My needs for intimacy, companionship, *etc*., could easily be fulfilled,” “If I were not dating my partner, I would do fine-I would find another appealing.”); Investment Size measures the degree of personal involvement in the intimate relationship (“I have put a great deal into our relationship that I would lose,” “I feel very involved in our relationship-like I have put a great deal into it.”). Moreover, Commitment level measures the extent to which a person is committed to developing the intimate relationship (“I am committed to maintaining my relationship with my partner,” “I am oriented toward the long-term future of my relationships.”). The participants and their partners indicated their responses on a 9-point Likert-type scale, ranging from 0 = strongly disagree to 8 = strongly agree. The higher scores indicated higher levels of Satisfaction (α = 0.83), Quality of Alternatives (α = 0.81), Investment Size (α = 0.74), and Commitment level (α = 0.78), respectively.

#### Measure of Stress-Alleviating Effect and Arousal Reduction in No-Touch Condition and Touch Condition

The participants' ratings of unpleasantness (arousal ratings) in alone condition were set as the baseline. The stress alleviation (the arousal reduction) for partner touch condition was calculated as the difference between the participants' ratings of unpleasantness (arousal ratings) in alone condition and the ratings in partner touch condition. The stress-alleviating effect (arousal reduction) for partner no-touch condition was calculated as the difference between the participants' ratings of unpleasantness (arousal) in alone condition and the ratings in partner no-touch condition. The bigger the difference of the ratings was, the stronger the stress-alleviating effect (the reduction of arousal) was.

#### Touch-Induced Stress-Alleviating Effect

Touch-induced stress-alleviating effect was the difference between the participants' ratings of unpleasantness in partner no-touch condition and the ratings in partner touch condition. The bigger the difference between the ratings was, the stronger the touch-induced stress-alleviating effect was.

### Statistical Analysis

Statistical analyses were carried out with the SPSS software (version 25.0). The continuous variables of interest were normally distributed, no transformations were necessary. We assessed the effect of partner touch on the stress alleviation and the arousal reduction by conducting a cue-type (threat, safety) × state (partner touch, partner no-touch) repeated measures ANOVA. Main effects and simple effects were tested *via* repeated measures ANOVA and paired *t*-test. Effect sizes were presented as partial eta-squared (η^2^ partial) and *p*-value.

Pearson correlation was used to examine the correlation between perceived relationship quality of couple-members (participants perceiving the quality of alternatives and the partners' commitment level) and touch-induced stress alleviation. Multiple linear regression was used to explore the important indicators in influencing touch-induced stress-alleviating effect. The level of significance was set at *p* < 0.05.

## Results

### Stress-Alleviating Effect of the Partner's Touch

**Figure 2:**
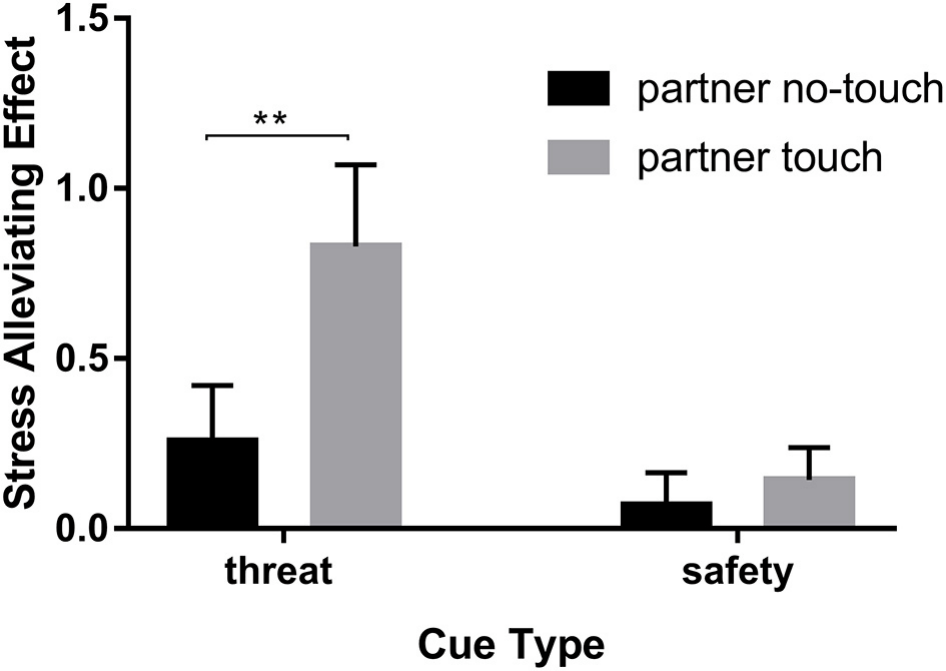
The stress-alleviating effect of the partner's touch. **p < 0.01.

We operated a series of repeated measures ANOVA to test our hypothesis. To examine the role of close partner's touch in alleviating stress, a 2 (cue-type: threat, safety) × 2 (state: partner touch, partner no-touch) repeated measures ANOVA on the stress-alleviating effect was conducted. As expected, the main effects of cue-type were found, *F*_(1,60)_ = 4.61, *p* < 0.05, ${\text{η}}_{\text{p}}^{2}$ = 0.07, and the stress-alleviating effect under threat (*M*_threat_ = 0.54, *SE* = 0.18) is significantly higher than that under safety (*M*_safety_ = 0.11, *SE* = 0.09, *t* = 0.437, *p* < 0.05). The main effects of state were significant, *F*_(1,60)_ = 8.83, *p* < 0.01, ${\text{η}}_{\text{p}}^{2}$ = 0.13, and the stress-alleviating effect of partner touch (*M*_partnertouch_ = 0.49, *SE* = 0.13) is significantly higher than that of partner no-touch (*M*_partnerno−touch_ = 0.16, *SE* = 0.09, *t* = 0.323, *p* < 0.01). There was also a significant cue-type × state interaction, *F*_(1,60)_ = 9.56, *p* < 0.01, ${\text{η}}_{\text{p}}^{2}$ = 0.14 (see Figure 2). The result of simple effects revealed that the participants who received touch from the partners (*M*_partner_ _touch_ = 0.83, *SE* = 0.24) have stronger stress-alleviating effect than the participants who received no touch (*M*_partner_ _no−touch_ = 0.26, *SE* = 0.16, *t* = 3.47, *p* < 0.01) when facing a threat. When facing safety, the stress-alleviating effect was not different between two states (*M*_partner_ _no−touch_ = 0.07, *SE* = 0.09; *M*_partner_ _touch_ = 0.14, *SE* = 0.09, *t* = −0.76, *p* > 0.05). The interaction may have been driven by the significantly higher stress-alleviating effect for partner touch state than partner no-touch state while facing a threat, confirming that affectionate touch of close partner promotes stress-alleviating effect effectively.

### Arousal Reduction of the Partner's Touch

**Figure 3:**
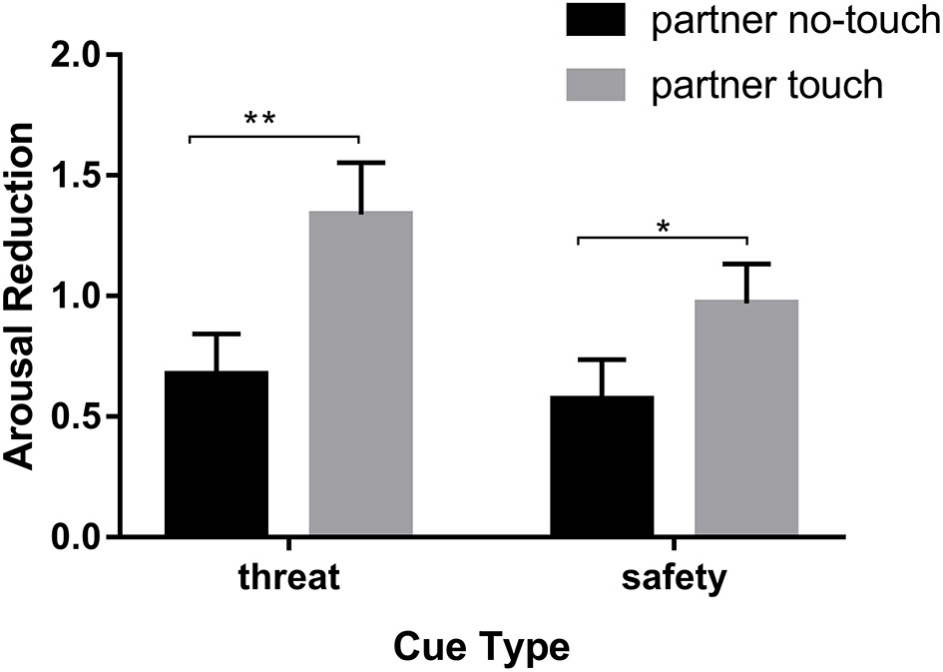
Arousal reduction of the partner's touch. *p < 0.05, **p < 0.01.

A 2 (cue-type: threat, safety) × 2 (state: partner touch, partner no-touch) repeated measures ANOVA on arousal reduction was conducted for the purpose of investigating the arousal reduction of the partner's touch. The main effects of state were not significant, *F*_(1,60)_ = 1.71, *p* > 0.05, ${\text{η}}_{\text{p}}^{2}$ = 0.028. The main effects of cue-type were significant, *F*_(1,60)_ = 8.98, *p* < 0.01, ${\text{η}}_{\text{p}}^{2}$ = 0.13, and arousal reduction under threat (*M*_threat_ = 1.15, *SE* = 0.16) was significantly higher than that under safety (*M*_safety_ = 0.62, *SE* = 0.14, *t* = 0.531, *p* < 0.01). There was a marginal significant cue-type × state interaction, *F*_(1,60)_ = 3.46, *p* = 0.068, ${\text{η}}_{\text{p}}^{2}$ = 0.054 (see Figure 3). The result showed that the touch-induced arousal reduction effect was stronger while facing a threat. This indicated that emotional valence (unpleasantness) was more sensitive for indicating emotion than emotional arousal (agitation).

### Perceived Relationship Quality of the Couple Modulating Touch-Induced Stress-Alleviating Effect

**Figure 4:**
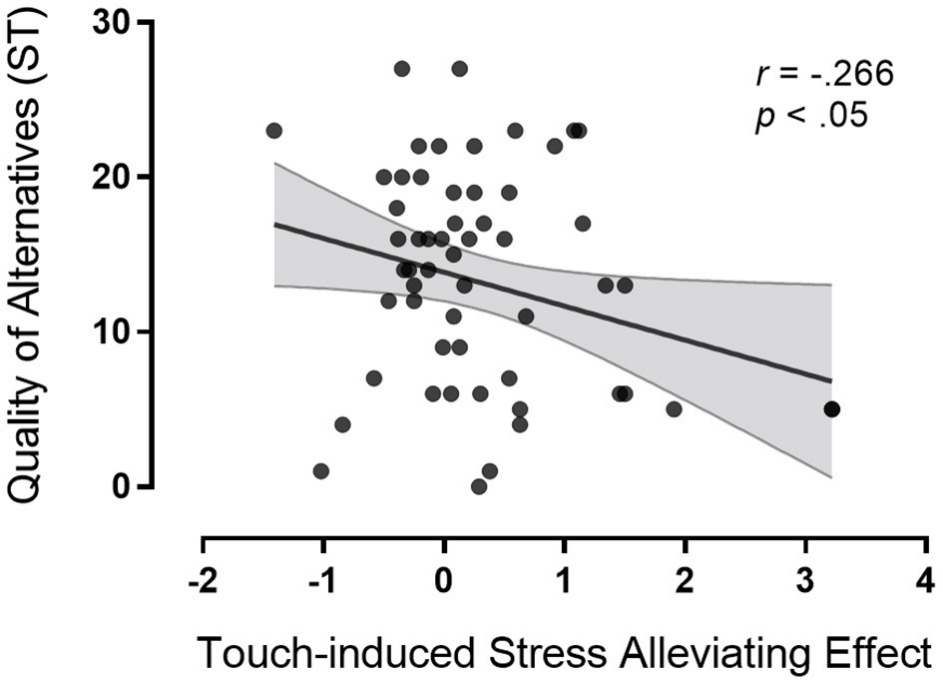
Pearson correlation and scatterplots for perceived quality of alternatives and touch-induced stress-alleviating effect.

**Figure 5:**
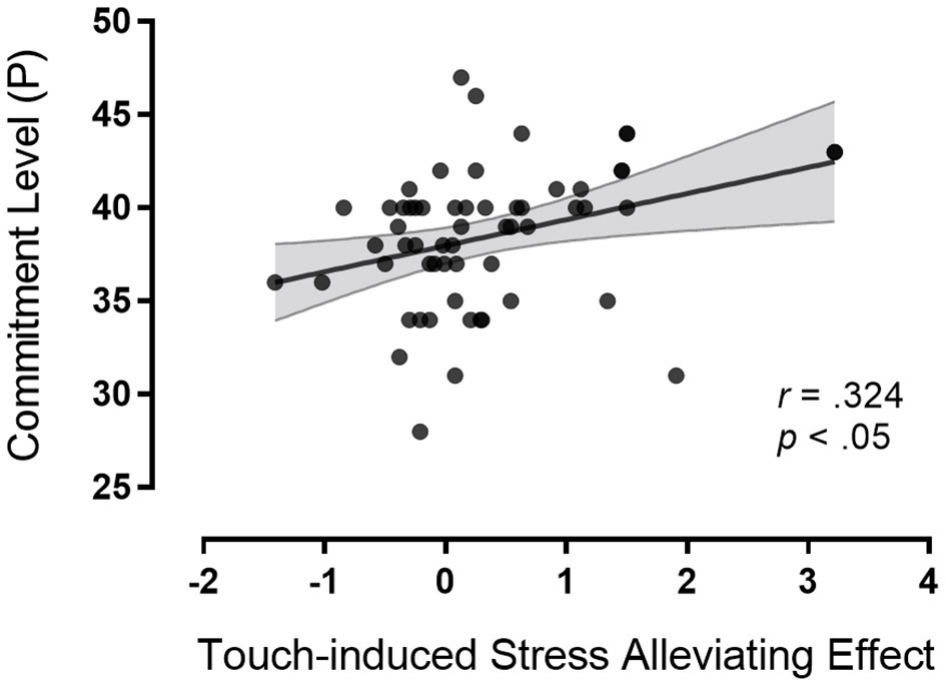
Pearson correlation and scatterplots for commitment level of the partner and touch-induced stress-alleviating effect.

We hypothesized that touch-induced stress-alleviating effect would be associated with the relationship quality of the couples, with high relationship quality predicting greater touch-induced stress alleviation. A significant negative correlation was found between participants perceiving the quality of alternatives and touch-induced stress alleviation (*r* = −0.266, *p* < 0.05). A significant positive correlation was found between partner's commitment and touch-induced stress alleviation (*r* = 0.324, *p* < 0.05) (see Figures 4, 5). Multiple linear regression was used to explore the degree to which perceived relationship of the couple-members (participants perceiving the quality of alternatives and the partner's commitment level) predicted touch-induced stress-alleviating effect.

We found that the perceived relationship quality of the couple-members (participants perceiving the quality of alternatives and the partner's commitment level) modulated touch-induced stress alleviation. Participants perceiving the low quality of alternatives and the high partners' commitment level showed stronger touch-induced stress-alleviating effect than participants perceiving the high quality of alternatives and the low partners' commitment level. The findings indicated that the stress-alleviating effect of touch was affected by actor and partner effects. The explained variance was around 16.8% jointly for actor and partner effects (*R*^2^ = 16.8%, see Table 1). Moreover, we found that the stress-alleviating effect of the presence of the partner with no touch was only correlated with participants perceiving the quality of alternatives (*r* = −0.285, *p* < 0.05). Compared with participants perceiving the high quality of alternatives, participants perceiving the low quality of alternatives showed stronger stress alleviation.

**Table 1:** Relationship quality of the couples modulating touch-induced stress-alleviating effect.

	**Partner's touch-induced stress-alleviating effect**	
**Predictor**	**B**	***P***
Commitment level (P)	0.072 [0.017, 0.128]	0.012^*^
Quality of Alternatives (ST)	−0.031 [−0.060, 0.004]	0.038^*^

*ST, stimuli-target; P, partner; B, unstandardized beta weights. 95% confidence intervals for significant effects are shown in brackets*.

* *p < 0.05*.

## Discussion

Whether the close partner's touch can serve as a strong resource to alleviate individual stress to the threat? Whether the touch-induced stress-alleviating effect is modulated by the relationship quality of the couples? What are the important factors of relationship quality influencing the effect of touch-induced stress alleviation? The study aimed to answer these questions by an electrical stimulation experiment. The results revealed that the close partner's touch could alleviate individual stress effectively. The relationship quality of the couple (participants perceiving the quality of alternatives and the partner's commitment level) could predict up to 16.8% of the variance in touch-induced stress alleviation.

The finding that the participants who received a touch from the partners had a stronger stress-alleviating effect than the participants who received no touch was consistent with existing studies. In these studies, the partners' touch had a significant stress alleviation effect. However, these studies focused primarily on comparing touch-induced stress-alleviating effect between different social relationships (Coan et al., 2017; Morriss et al., 2019). The current experiment extended the previous research studies by comparing touch-induced stress-alleviating effect in different qualities of romantic relationships. Here, we showed evidence supporting the stress-alleviating effect of partner's touch and an impact of relationship quality on the stress alleviation of touch. Specifically, we demonstrated that the stress-alleviating effect of touch was affected by actor and partner effects (i.e., participants perceiving the low quality of alternatives and the high partners' commitment level showed stronger touch-induced stress-alleviating effect than participants perceiving the high quality of alternatives and the low partners' commitment level). As a non-verbal straightforward interactive way in emotional communication (Gallace and Spence, 2010), affectionate touch may be a bridge sharing dyadic interpersonal interaction process. Exploring touch-induced stress-alleviating effect from a two-way perspective will contribute to our understanding of affection interaction in an intimate relationship.

It has been proposed that perceived partner commitment was one of the most reliable factors predicting relationship quality and stability of relationship (Joel et al., 2020). Consistent with this proposition, we showed that the commitment of the close partner predicted the effect of touch-induced stress alleviation, suggesting a potential essence of love. Partners' commitment might increase individuals' social resources and enhance individuals' perceived support, thus having more confidence while facing a threat.

Some researchers examined the influence of affectionate touch on individuals' well-being and social relationship (Hertenstein et al., 2006b; Kim et al., 2018). Recently, Jakubiak and Feeney proposed a theoretical mechanistic model that affectionate touch might promote positive changes of relationship cognition, which reduce stress indirectly by enhancing relational, psychological, and physical well-being (Jakubiak and Feeney, 2019). However, the theoretical model neglects the interaction between the relationship quality and the behavior of touch. On the one hand, positive touch can promote relationship quality; on the other hand, relationship quality can influence the outcome of touch. Thus, our findings extend the recent theory explaining the theoretical mechanistic model of the stress-alleviating effect of touch and improve the theoretical model to fully explain why touching relieves stress. Specifically, we observed that the relationship quality of couples modulated the touch-induced stress alleviation.

The results indicated that the touch of the partners made the participants feel more secure under threat condition than the presence of the partners with no touch. In a digital immersive virtual environment study, Kane et al. found that the presence of the attentive partner made the participant feel more secure in a threatening cliff-walking task than that of the inattentive partner (Kane et al., 2012). Combined with the two studies, it will be significant for future research to explore the psychological mechanisms of different ways of social interaction-induced stress-alleviating effects.

Although the current research makes theoretical and practical contributions, it has several limitations that need to be acknowledged. First, causality between the perception of the relationship quality of both partners and affectionate touch-induced stress alleviation should be considered with caution because of the correlational design of the current study. Although the causal inferences are limited by the correlational design, exploring the stress-alleviating effect of close partner's touch from dualistic perspective has its benefits. It is worth noting that the current study offers evidence for the value of a dualistic perspective on social interaction research. Second, data analysis of the study relies on the participants' self-reports, although the rating methods are often used in emotion-related studies. Thus, the objective indicator should be used in further research. Third, the research is limited by the characteristic of the sample, and participants are relatively young and unmarried couples. Compared with unmarried couples, married couples may have the emotion-rich experience, so more work needs to be done to verify the external validity of the results.

Taking these limitations into consideration, the robust causality experiment should be conducted. Moreover, future research should test moderator variables other than the perceived relationship quality of couple-members from a dualistic perspective. As one of the most fundamental communication ways, touch is associated with many physical and psychological outcomes for the couple, such as daily stress (Burleson et al., 2007), mood (Ditzen et al., 2008), and relationship satisfaction (Gulledge et al., 2003). Therefore, it is important for researchers to explore the active influencing factors of touch-induced positive effect.

## Data Availability Statement

The raw data supporting the conclusions of this article will be made available by the authors, without undue reservation.

## Ethics Statement

The studies involving human participants were reviewed and approved by the Ethics Committee of University of Science and Technology of China (Date: 2020-05-26/No. 2020KY78). The patients/participants provided their written informed consent to participate in this study.

## Author Contributions

DL, HS, and XZ conceived and designed the study. LZ, RM, and YZ recruited the participants and collected the data. WG supervised the data collection. YP and WL analyzed the data. DL and HS wrote the manuscript. All authors contributed to the article and approved the submitted version.

## Conflict of Interest

The authors declare that the research was conducted in the absence of any commercial or financial relationships that could be construed as a potential conflict of interest.
